# Selected Dietary and Supplemental Phytochemicals in Adiposity-Based Chronic Disease: Molecular Mechanisms, Human Evidence, and Translational Gaps

**DOI:** 10.3390/nu18213630

**Published:** 2026-10-31

**Authors:** Arguiñe Ivonne Urraza-Robledo, Edmar de Jesús Díaz García, Ibzan Jahzeel Salvador Ibarra, Zoila Mora Guzmán, Anahí Jobeth Borrás Enríquez, Eduardo Pérez-Campos, Laura Pérez-Campos Mayoral, Daniel Gómez Hernández, María del Socorro Pina-Canseco, Hector Alejandro Cabrera-Fuentes, María Teresa Hernández-Huerta

**Affiliations:** 1 Centro de Mezclas Oncológicas, Unidad Médica de Alta Especialidad No. 71, Instituto Mexicano del Seguro Social, Coahuila de Zaragoza 27000, Mexico; ivonne_urraza@hotmail.com; 2 Centro de Investigación Facultad de Medicina UNAM-UABJO, Facultad de Medicina y Cirugía, Universidad Autónoma “Benito Juárez” de Oaxaca, Oaxaca 68020, Mexico; edg.89@live.com.mx (E.d.J.D.G.); zmora.fmc@uabjo.mx (Z.M.G.); anajoborras@gmail.com (A.J.B.E.); lperez.cat@uabjo.mx (L.P.-C.M.); danielgomez25@outlook.es (D.G.H.); mpina.cat@uabjo.mx (M.d.S.P.-C.); 3 Instituto Mexicano del Seguro Social, Hospital General de Zona con Medicina Familiar No. 18, Coahuila de Zaragoza 27220, Mexico; ibzjah@gmail.com; 4 Secretaría de Ciencia, Humanidades, Tecnología e Innovación (SECIHTI), Departamento de Farmacología, Facultad de Medicina, Universidad Autónoma de Coahuila, Coahuila de Zaragoza 25000, Mexico; 5 Secretaría de Ciencia, Humanidades, Tecnología e Innovación (SECIHTI), Facultad de Medicina y Cirugía, Universidad Autónoma “Benito Juárez” de Oaxaca, Oaxaca 68020, Mexico; 6 División de Estudios de Posgrado e Investigación, Tecnológico Nacional de México, Instituto Tecnológico de Oaxaca, Oaxaca 68030, Mexico; pcampos@itoaxaca.edu.mx; 7 R&D Group, Vice Presidency for Scientific Research and Innovation, Imam Abdulrahman Bin Faisal University, Dammam 31441, Saudi Arabia; 8 División de Estudios de Posgrado e Investigación, Tecnológico Nacional de México, Instituto Tecnológico de Tijuana, Tijuana 22414, Baja California, Mexico

**Keywords:** adiposity-based chronic disease, obesity, phytochemicals, quercetin, chlorogenic acids, epigallocatechin-3-gallate, berberine, adipogenesis, thermogenesis, body composition

## Abstract

Adiposity-based chronic disease (ABCD) encompasses the health consequences of excess or dysfunctional adipose tissue. Selected phytochemicals have been investigated as potential adjuncts, but experimental pathway activity does not establish clinical benefit. This narrative review examines quercetin, chlorogenic acids, epigallocatechin-3-gallate (EGCG), and berberine, with other interventions providing context. The evidence was supplemented by targeted PubMed searches through 27 October 2026 and appraised according to experimental model, formulation, exposure, clinical relevance, and safety. Published pooled body-weight differences were −0.35 kg (95% confidence interval, −2.03 to 1.33) for quercetin, −1.30 kg (−2.07 to −0.52) for selected green-coffee/chlorogenic-acid interventions, −0.74 kg (−0.97 to −0.51) for green-tea preparations, and −0.88 kg (−1.36 to −0.39) for berberine. These source-specific estimates are not head-to-head comparisons. A small crossover trial of quercetin reported reduced hepatic fat, whereas a six-month berberine trial found no significant benefit for visceral or hepatic fat. Coffee and green-tea findings do not isolate the effects of chlorogenic acid or EGCG. Most mechanistic evidence remains preclinical, and differences in chemical composition, circulating metabolites, administration, and outcome selection limit translation. The human-evidence-based selection also underrepresents less-studied phytochemicals; for the evaluated interventions, sustained adiposity reduction and patient-relevant benefit remain insufficiently established. Their potential role in comprehensive ABCD care requires product-specific assessment of efficacy, exposure, adverse effects, and interactions.

## 1. Introduction

The adiposity-based chronic disease (ABCD) framework describes the adverse health consequences of excess or dysfunctional adipose tissue and emphasizes disease severity, complications, and individual treatment goals. Contemporary guidance assesses adiposity alongside medical, functional, and psychological impairment rather than relying on body mass index (BMI) alone [1,2]. The 2025 Commission on clinical obesity further distinguishes excess adiposity with current organ dysfunction or limitations in daily activities from preclinical obesity, in which organ function is preserved despite increased future risk [3]. These frameworks are related but not interchangeable. We use ABCD as the clinical context and retain obesity, overweight, and the diagnostic labels reported in individual studies to preserve the identity of their populations and outcomes.

Excess and dysfunctional adipose tissue are associated with type 2 diabetes (T2D), cardiovascular disease, metabolic dysfunction-associated steatotic liver disease (MASLD), disability, and several cancers [4]. Global projections indicate that overweight and obesity will continue to rise if historical trends persist [5]; BMI remains useful for surveillance and initial assessment, but it does not distinguish visceral or ectopic fat, lean mass, or the complications that determine an individual’s clinical needs [2,3].

The pathophysiology of ABCD extends beyond sustained positive energy balance and involves complex alterations in central and peripheral appetite regulation [6]; it includes adipose tissue expansion through adipocyte hypertrophy and hyperplasia, mitochondrial dysfunction, ectopic lipid deposition, insulin resistance, extracellular matrix remodeling, and chronic low-grade inflammation [2,7]. Alterations in gut microbial functions and intestinal barrier integrity may further contribute to obesity-associated metabolic dysfunction and systemic inflammation [8,9]. These processes differ across individuals and disease stages, helping explain why treatment response varies and why a single biomarker or pathway rarely captures the full phenotype.

Comprehensive care combines nutrition, physical activity, behavioral and psychological support, and, when appropriate, pharmacotherapy or metabolic procedures [1,2,10,11]. Randomized trials demonstrate substantial average weight reduction with semaglutide and tirzepatide, and a head-to-head trial found greater weight reduction with tirzepatide than with semaglutide in the tested population [12,13,14]; these clinical comparisons do not identify the relative contribution of individual molecular mechanisms. Access and affordability, gastrointestinal adverse effects, and weight regain after treatment withdrawal remain relevant limitations [11,15,16]. They provide context for investigating adjunctive approaches, but neither botanical origin nor the number of proposed molecular targets establishes superior efficacy or safety [11,17].

Plant-derived phytochemicals encompass diverse chemical classes, including polyphenols, alkaloids, terpenoids, saponins, carotenoids, and phytosterols [18,19]. Studies of selected compounds in experimental systems have reported effects on digestive enzyme activity, appetite-related signaling, adipocyte differentiation and lipid metabolism, energy-sensing pathways, inflammatory and redox responses, and gut microbiota composition and function [18,19,20]. However, calling phytochemicals “multi-target” agents requires careful interpretation, as changes in molecular pathways do not necessarily indicate direct target engagement or causal mechanisms [21,22]. Also, concentrations used in experimental models may not reflect physiologically achievable human exposure, and observed molecular responses do not necessarily translate into clinically meaningful reductions in adiposity [21,23,24].

Therefore, this review examines tissue-specific mechanisms, compares experimental findings with human exposure and clinical outcomes, and identifies the remaining translational gaps for four selected compounds: quercetin, chlorogenic acids (CGAs), epigallocatechin-3-gallate (EGCG), and berberine; they are illustrative cases with different chemical and pharmacological profiles, not a validated ranking or a list of established treatments. The distinction between dietary and supplemental exposure is central to this scope: quercetin, CGAs, and tea catechins occur in commonly consumed foods or beverages, whereas the berberine interventions evaluated here use concentrated oral preparations, including berberine hydrochloride [25,26,27,28,29]. Berberine is included as a supplemental alkaloid with human trials and pharmacokinetic information, not as a representative constituent of the usual diet; food matrices, botanical extracts, and isolated compounds are therefore evaluated separately [24,30,31]. The central translational question is whether the reported mechanisms remain clinically relevant when the tested preparation, human exposure, and adiposity-related outcomes are considered together.

## 2. Scope and Approach of the Narrative Review

This critical narrative review examines experimental and human evidence on selected dietary and supplemental phytochemicals in adiposity-based chronic disease. Quercetin, CGAs, EGCG, and berberine were selected for their defined chemical identity and the availability of mechanistic research, human studies, and exposure or safety data; their inclusion does not constitute a ranking of therapeutic efficacy. Literature was identified through exploratory searches in PubMed and Google Scholar, followed by a PubMed update completed on 27 October 2026. The update covered publications from 1 January 2000 to 27 October 2026, without language restrictions. Each clinical query used two conceptual blocks: (1) condition- and outcome-related terms, including obesity, overweight, adiposity, weight loss, body weight, body mass index, BMI, waist circumference, visceral fat, fat mass, and liver fat; and (2) compound, botanical, or preparation names. Terms within each block were combined with OR, and the two blocks with AND. Four main queries used different terms in the second block: quercetin; chlorogenic acid(s), coffee, green coffee, artichoke, cardoon, and *Cynara cardunculus*; EGCG, epigallocatechin gallate, epigallocatechin-3-gallate, green tea, and *Camellia sinensis*; and berberine. Publication-type filters covered clinical trials, randomized controlled trials, systematic reviews, and meta-analyses. These queries retrieved 61, 207, 218, and 63 records, respectively; its counts refer to records retrieved before selection, may overlap across queries, and do not represent numbers of included studies.

Separate queries addressed capsaicin, saffron, anthocyanins, phytosterols, and sulforaphane; a further query covered foods and plant by-products as sources of CGAs. These searches supplemented the four main clinical queries rather than adding mandatory search blocks. Targeted searches and reference-list examination identified additional experimental, pharmacokinetic, chemical-composition, and methodological literature. Limitations related to search coverage and incomplete documentation of the original searches are discussed in Section 9. Articles were selected by the co-first authors (A.I.U.-R. and E.J.D.G.) and corresponding authors (H.A.C.-F. and M.T.H.-H.) according to their relevance to the review’s scope; disagreements regarding inclusion were resolved through discussion and consensus. Both favorable and neutral human findings were interpreted according to preparation, population, dose, duration, and outcome. Cellular and animal findings were assessed within their experimental context, distinguishing direct binding and functional perturbation evidence from changes in gene expression or phosphorylation, molecular docking predictions, and omics associations. Estimates from published meta-analyses were reported with their original confidence intervals.

## 3. Clinical Care for Adiposity-Based Chronic Disease and the Role of Phytochemicals and the Appropriate Role of Phytochemicals

ABCD care addresses nutrition, enjoyable and feasible physical activity, sleep, psychological well-being, weight-related stigma, functional limitations, and treatment access. Medical complications, daily functioning, quality of life, and the individual’s priorities contribute to treatment selection and follow-up [1,2,11]. Weight and body-composition changes are relevant outcomes, but health improvement cannot be reduced to the number on the scale. The substantial clinical effects of incretin-based therapies provide a useful evidence standard for adjunctive interventions [12,13,14]; their actions are already biologically complex, so a contrast between multi-target natural products with single-target drugs would be misleading. For phytochemicals, the relevant question is whether a characterized preparation produces a reproducible benefit, at a tolerable exposure, that adds value within comprehensive care [17,23,30].

The intended use changes the evidence needed for a phytochemical. Potential uses include a component of a food-based prevention pattern; a chemically standardized supplement with a modest metabolic effect; an adjunct to dietary, behavioral, or pharmacological treatment; a molecular probe for pathway discovery; or a scaffold for medicinal-chemistry development [32,33]; these are possible research applications, not established indications. Food-derived exposures can be evaluated through dietary exposure, food-matrix effects, historical consumption patterns, and available long-term safety data [30,34]. In contrast, concentrated botanical extracts, particularly those intended for therapeutic use, warrant rigorous characterization of their chemical composition, standardization, dose–exposure relationships, pharmacokinetics, potential herb–drug interactions, and adverse events [31,34,35].

## 4. Tissue-Specific Molecular Pathways

**Figure 1:**
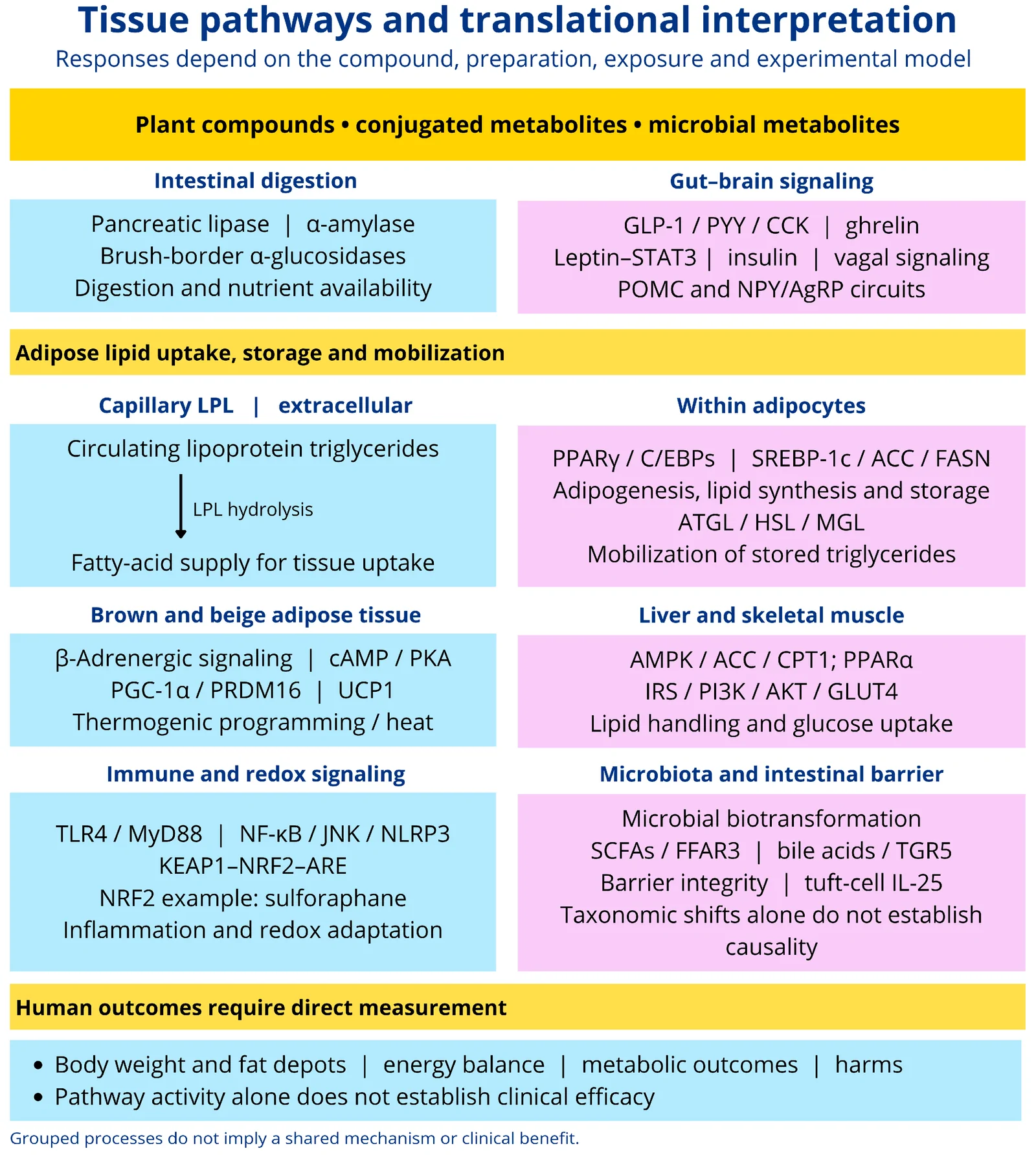
Tissue-specific processes relevant to selected dietary and supplemental phytochemicals [42,43,44,45]; the corresponding pathways and evidence are detailed in Section 4. Abbreviations: ACC, acetyl-CoA carboxylase; AgRP, agouti-related peptide; AMPK, AMP-activated protein kinase; ARE, antioxidant response element; ATGL, adipose triglyceride lipase; C/EBP, CCAAT/enhancer-binding protein; CPT1, carnitine palmitoyltransferase 1; FASN, fatty-acid synthase; FFAR3, free fatty-acid receptor 3; GLP-1, glucagon-like peptide-1; GLUT4, glucose transporter 4; HSL, hormone-sensitive lipase; KEAP1, Kelch-like ECH-associated protein 1; LPL, Lipoprotein lipase; MGL, monoacylglycerol lipase; NF-κB, nuclear factor kappa B; NRF2, nuclear factor erythroid 2-related factor 2; PGC-1α, PPARγ coactivator 1α; PPAR, peroxisome proliferator-activated receptor; SCFAs, short-chain fatty acids; SREBP-1c, sterol regulatory element-binding protein 1c; UCP1, uncoupling protein 1.

Figure 1 maps the proposed, model-dependent actions of selected phytochemicals by tissue and biological process. The framework emphasizes that orally ingested compounds first encounter the food matrix, digestive tract, intestinal metabolism, and microbiota. Parent compounds, conjugated metabolites, and microbiota-derived metabolites may influence intestinal nutrient digestion and absorption [24,36], gut–brain appetite regulation [37], adipocyte differentiation and lipid metabolism [20], thermogenesis in brown and beige adipose tissue [20,38], intestinal barrier integrity and microbial metabolism [24,38,39], and inflammatory signaling and cellular redox homeostasis [20,39]. Additional effects on hepatic and skeletal muscle metabolism may involve pathways regulating fatty-acid oxidation, lipogenesis, insulin signaling, and glucose uptake [20,40]. These experimentally observed mechanisms may contribute to changes in energy intake and expenditure, adiposity, ectopic lipid accumulation, insulin sensitivity, and systemic inflammation; however, their translation into clinically meaningful outcomes remains uncertain [23,41]. The map combines physiological background and intervention-specific examples; it does not imply that all compounds affect every pathway or that the connections have been established in humans.

### 4.1. Gastrointestinal Digestion and Nutrient Availability

Selected plant compounds and extracts inhibit pancreatic lipase, α-amylase, or intestinal α-glucosidases in experimental systems [46,47,48]; these luminal or brush-border targets do not necessarily require appreciable plasma exposure. However, relevance depends on local dose, stability during digestion, the food matrix, and the enzyme system used [48,49,50]. Quercetagetin, for example, inhibited human intestinal α-glucosidases in vitro, but this does not establish a benefit for human adiposity [48]. Acute studies of mulberry-derived preparations show measurable effects on carbohydrate handling under specified conditions [51,52,53]; such postprandial responses do not demonstrate sustained weight loss. Similarly, a 12-week green-tea-extract trial found no significant change in fecal fat or energy excretion, or body composition [54]. The distinction between a biochemical inhibition signal, a physiological absorption effect, and a durable clinical outcome is therefore essential.

### 4.2. Gut–Brain Signaling and Appetite Regulation

Gastrointestinal nutrient sensing communicates with vagal afferents, brainstem circuits, and hypothalamic networks [55,56]. Leptin and insulin contribute to energy-homeostatic signaling, whereas ghrelin, glucagon-like peptide-1 (GLP-1), peptide YY (PYY), and cholecystokinin (CCK) participate in hunger, satiation, or satiety [57,58,59,60,61]. These physiological mechanisms provide context; they do not directly show that the four focal compounds engage the same circuits in humans. A contemporary CCK synthesis supports satiation effects but does not establish long-term weight-loss efficacy [61].

Oral grape-seed proanthocyanidin extract altered hypothalamic leptin/STAT3 signaling and neuropeptide expression in diet-induced obese rats, without reversing body-weight gain [62]. Other experimental studies support enteroendocrine or neural hypotheses under defined administration conditions [63,64]. Changes in food intake can also reflect palatability, aversion, locomotor changes, or hydration, rather than a specific satiety mechanism [65,66,67,68]. Pair-feeding can help separate some intake-related effects, although ration matching has analytical and biological limitations [69]. Human appetite ratings, observed intake, and subsequent weight outcomes likewise measure different constructs [70,71].

### 4.3. Adipogenesis and De Novo Lipogenesis

Adipocyte differentiation involves an ordered transcriptional program in which CCAAT enhancer-binding protein (C/EBP) and peroxisome proliferator-activated receptor γ establish the mature adipocyte phenotype [72]. Sterol regulatory element-binding protein 1c promotes expression of lipogenic enzymes such as acetyl-CoA carboxylase (ACC) and fatty-acid synthase (FASN) [73]. Selected phytochemicals reduce lipid accumulation in 3T3-L1 cells and are associated with lower peroxisome proliferator-activated receptor γ (PPARγ), C/EBPα, sterol-regulatory element-binding protein 1c (SREBP-1c), ACC, or FASN expression [74,75]. PPARγ is not simply a harmful adipogenesis switch; appropriate PPARγ activity supports insulin-sensitive adipocytes and safer lipid storage [76,77]. The PPARγ-LPCAT3 axis has been identified as a mechanistic link between dietary intake of *n*-6 polyunsaturated fatty acids (PUFAs), adipose tissue expansion capacity, and systemic energy balance [77]. Suppressing differentiation in vitro may be irrelevant or even unfavorable if it promotes hypertrophic, dysfunctional adipocytes or ectopic lipid deposition in vivo [77,78]. Interpretation is stronger when studies link transcriptional changes to adipocyte size and number, insulin responsiveness, tissue fibrosis, circulating fatty acids, and hepatic or muscular lipid accumulation [76,77,78].

### 4.4. Lipolysis, Fatty-Acid Oxidation, and Hepatic Metabolism

Intracellular triglyceride mobilization involves adipose triglyceride lipase (ATGL), hormone-sensitive lipase (HSL), and monoacylglycerol lipase (MGL) [42]. By contrast, capillary lipoprotein lipase (LPL) hydrolyzes circulating lipoprotein triglycerides to support tissue fatty-acid delivery; experimental adiponectin signaling can increase adipose LPL activity and triglyceride uptake [43]. Neither process is equivalent to pancreatic lipase-mediated dietary digestion; this distinction prevents the addition of LPL to Figure 1 from implying that all lipases share the same location or therapeutic effect. Fatty-acid oxidation depends on mitochondrial transport and capacity, including the AMP-activated protein kinase (AMPK)/ACC/malonyl-CoA/carnitine palmitoyl transferase 1 (CPT1) axis [79]. Individual compounds have been linked to AMPK/ACC and PPARα-associated responses in experimental systems [80,81], but the systems differ, including liver-injury models that do not establish an anti-obesity effect. Phosphorylation of AMPK or HSL is not a direct measurement of whole-body fat oxidation [42,82,83]. Greater lipolysis without adequate oxidation or safe lipid storage can increase non-esterified fatty-acid delivery and ectopic lipid exposure [84,85]. Interpretation therefore depends on functional flux, tissue lipid content, insulin responsiveness, intake, and body composition alongside molecular markers [42,79,84].

### 4.5. Adaptive Thermogenesis and Adipose-Tissue Browning

Uncoupling protein 1 (UCP1)-dependent thermogenesis in brown and beige adipocytes involves proton conductance across the inner mitochondrial membrane, uncoupling substrate oxidation from ATP synthesis and enabling energy dissipation as heat [86]. Sympathetic and transcriptional mechanisms regulate thermogenic recruitment and activation, involving cyclic AMP, protein kinase A, p38 MAPK, PGC-1α, and PRDM16, as well as mitochondrial programming and UCP1 expression; these components do not necessarily form an obligatory linear pathway for every intervention [87,88]. Mouse studies of quercetin and berberine have reported increases in thermogenic markers (e.g., protein UCP-1 and deacetylation of PPARγ via AMPK/SIRT1) along with changes in energy expenditure [89,90]. In cultured brown adipocytes, a 2019 study reported that chlorogenic acid increased mitochondrial number, proton leak, and thermogenic activity; these findings provide cellular evidence rather than demonstrating increased whole-body thermogenesis in humans [91]. Rodent thermogenic responses depend substantially on the thermal environment. Housing below thermoneutrality increases thermoregulatory demand, and cold-induced increases in energy expenditure can be accompanied by compensatory increases in food intake [92]. Accordingly, housing and acclimation temperatures affect the interpretation of functional outcomes such as oxygen consumption and carbon dioxide production, estimates of substrate oxidation, core or tissue temperature, food intake, and body composition, alongside molecular markers [90,92]. The relevance to humans remains conditional on functional effects at documented human exposures. Imaging findings also require careful interpretation: ^18^F-fluorodeoxyglycose (^18^F-FDG) measured brown-adipose-tissue (BAT) glucose uptake can change independently of thermogenesis, so increased tracer uptake alone does not establish increased heat production [93].

### 4.6. Insulin Signaling and Glucose Homeostasis

Insulin-stimulated glucose uptake involves insulin receptor substrate–PI3K–Akt signaling and the mobilization of glucose transporter type 4 (GLUT4) to the plasma membrane in insulin-responsive tissues, including skeletal muscle and adipose tissue [43,94,95]. Obesity-associated inflammation, lipid intermediates, and mitochondrial redox disturbances can impair insulin responsiveness, although impaired glucose transport does not invariably coincide with reduced proximal Akt phosphorylation [94,95,96]. Experimental studies indicate that adiponectin can increase AMPK activation, promote fatty-acid oxidation, and improve insulin-stimulated glucose uptake in mice; these findings support biological plausibility but do not show the same magnitude of benefit in humans [43,97]. Changes in fasting glucose, fasting insulin, or homeostatic model assessment for insulin resistance (HOMA-IR) are metabolic or surrogate outcomes; they do not, by themselves, demonstrate restoration of a specific insulin-signaling defect [28,94,95]. Mechanistic assessment is strengthened by combining signaling measurements with GLUT4 translocation and glucose-uptake assays, supplemented where appropriate by hyperinsulinemic–euglycemic clamps and targeted loss-of-function experiments [43,94,95,97]. Glucose-tolerance testing provides an integrated physiological outcome but does not identify the molecular step responsible for a change [43,97]. These considerations separate metabolic effects from adiposity changes; a claim of weight-independent benefit needs a design or analysis capable of testing that relationship. A 2026 randomized trial in adults with obesity and MASLD without diabetes shows the need for direct clinical assessment: berberine at 1 g/day for six months did not reduce visceral adipose tissue (VAT) or liver fat compared with placebo, with no significant difference in adverse-event incidence during the randomized period after a run-in that excluded intolerance [28].

### 4.7. Adipose Inflammation and Redox Regulation

In experimental obesity, adipocyte enlargement, hypoxic stress, altered extracellular-matrix turnover, and adipocyte death can contribute to macrophage accumulation or activation [98,99,100]. Recruitment and activation of resident immune cells differ across models [96,99]. Toll-like receptor 4 (TLR4)/myeloid differentiation primary response 88 (MyD88)–inhibitor of nuclear factor kappa B kinase (IKK)/nuclear factor-kappa B (NF-κB), and nucleotide-binding domain and leucine-rich repeat-containing protein 3 (NLRP3) related signaling provide relevant inflammatory context, but no single sequence accounts for all interventions [96,101,102]. The Kelch-like ECH-associated protein 1 (KEAP1)–nuclear factor erythroid 2-related factor 2 (NRF2; encoded by *NFE2L2*) system regulates electrophilic and oxidative stress through antioxidant response elements (AREs) [44]. Sulforaphane is a relevant electrophilic inducer; its precursor glucoraphanin attenuated weight gain and metabolic dysfunction in high-fat-fed wild-type mice, whereas benefits were absent in NRF2-deficient mice [45]. This experiment justifies inclusion of the pathway and the *Brassica*-derived example, but does not establish human fat-loss efficacy; chemical radical-scavenging assays alone cannot demonstrate cellular NRF2 dependence [44]. Quercetin reduced adipose macrophage infiltration and pro-inflammatory markers in high-fat-diet-fed mice, accompanied by changes in AMPKα1/silent information regulator 1 (SIRT1) signaling [103]. Pharmacological experiments strengthened that model-level interpretation, although they did not establish genetic necessity in vivo or human target engagement. The quercetin and sulforaphane examples therefore support distinct experimental mechanisms rather than a common, clinically proven action.

### 4.8. Gut Microbiota, Intestinal Barrier, and Bile-Acid Signaling

Interactions among diet, the gut microbiota, and microbial metabolites can modify host energy availability and metabolic signaling [104]. Experimentally supported routes include short-chain fatty-acid sensing through vagal free fatty-acid receptor 3 (FFAR3) in mice [105], bile-acid-associated thermogenic responses in quercetin-treated mice [89], and microbial-metabolite-dependent regulation of colonic barrier and immune responses [106]. These routes represent context-dependent mechanisms rather than a single established causal sequence leading to human obesity [89,104,105,106]. In humans, a short randomized trial of green-tea extract reported improvements in selected intestinal-barrier and circulating endotoxin measures without a significant body-weight effect, illustrating the need to distinguish these outcomes from anti-obesity efficacy [107]. Some dietary phytochemicals undergo microbial biotransformation in the intestinal lumen, generating metabolites with distinct chemical structures, biological activities, and patterns of local or systemic exposure. Parent compounds and their microbial products can alter microbial growth and community composition [108]. Taxonomic associations alone do not establish a causal anti-obesity mechanism. In particular, the Firmicutes-to-Bacteroidetes ratio does not consistently reproduce an obesity signature across populations [109]. Mechanistic evaluation should connect metagenomic observations to measured metabolites, such as short-chain fatty acids (SCFAs) and bile acids, and to relevant host responses, including intestinal-barrier function [104,106,108]. Appropriately controlled germ-free or antibiotic-depletion studies, fecal microbiota transfer, and direct perturbation of candidate microbial enzymes, metabolites, or host receptors can strengthen causal attribution; these approaches provide complementary evidence and need not all be included in every study [89,105,108]. Human findings can reflect association, prediction, or mediation, and are affected by diet, medication exposure, geographical context, and baseline microbiota, all of which can influence the observed microbial or metabolic response [104,109,110,111]. For example, a secondary analysis of a clinical trial in adults with obesity and metabolic syndrome identified lower baseline *Prevotella* abundance as a candidate predictor of improvement in HOMA2-IR following fecal microbiota transplantation combined with fiber supplementation. Responders also exhibited greater engraftment of donor-derived *Faecalibacillus intestinalis* and *Roseburia* spp [111]. Table 1 summarizes these physiological pathways, which provide the context for the compound-specific appraisal in Section 5.

## 5. Focused Appraisal of Four Selected Compounds

**Figure 2:**
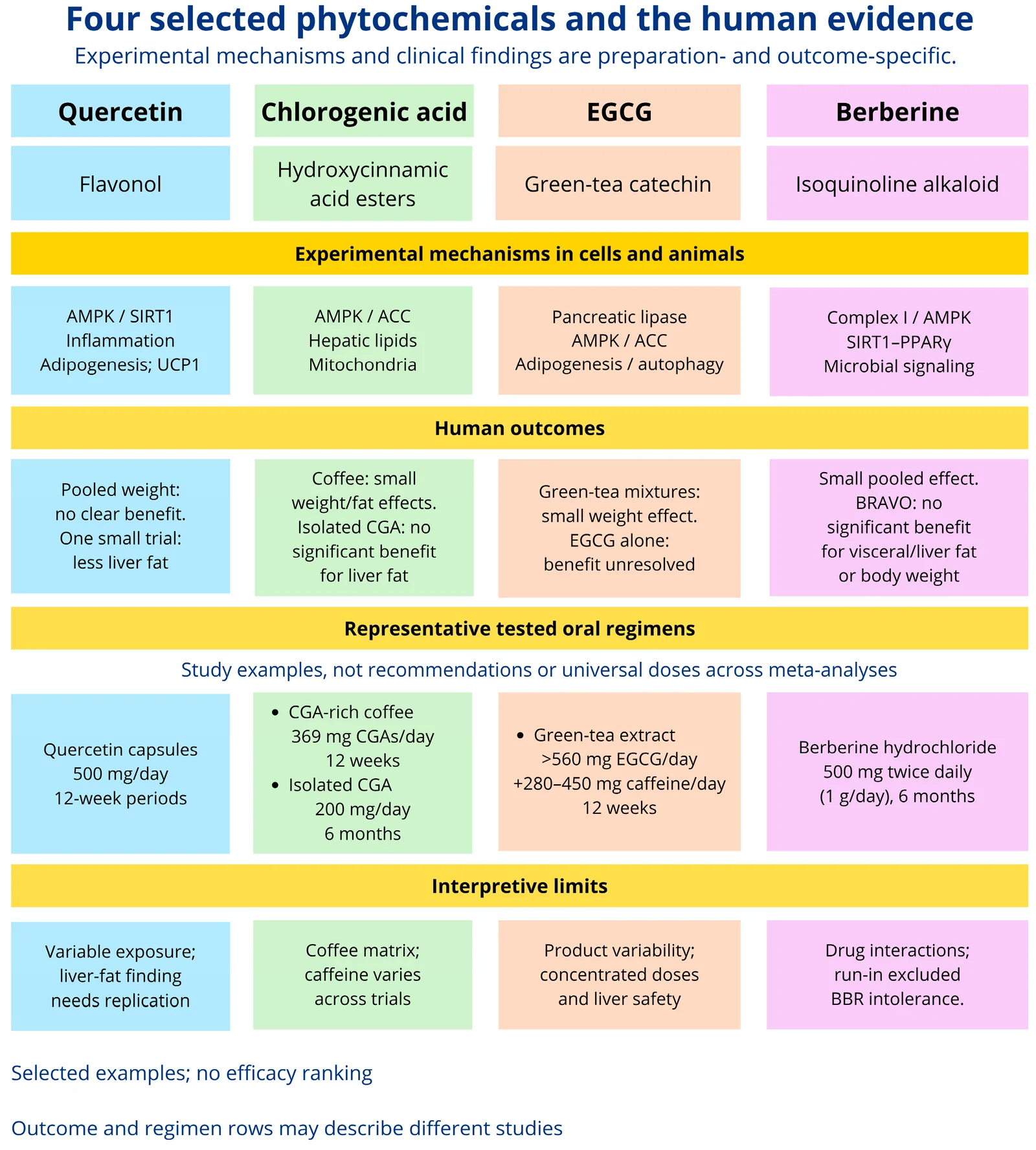
Comparison of the four selected compounds by experimental mechanism, human evidence, and representative tested oral regimen [28,54,112,113,114]. Abbreviations: ACC, acetyl-CoA carboxylase; AMPK, AMP-activated protein kinase; BBR, berberine; CGAs, chlorogenic acids; EGCG, epigallocatechin-3-gallate; PPAR, peroxisome proliferator-activated receptor; SIRT1, sirtuin 1; UCP1, uncoupling protein 1.

The four compounds in Figure 2 illustrate different challenges in connecting experimental mechanisms to human outcomes. Their selection reflects available mechanistic and human data, not comparative superiority. Figure 2 reports representative tested oral regimens; these are neither treatment recommendations nor a common dose for all trials in the corresponding meta-analyses.

### 5.1. Quercetin

Quercetin is a flavonol present in onions, apples, berries, and other plant foods [25]. Mouse and cellular studies implicate inflammatory regulation, lipophagy, and thermogenic programming [103,115,116]. In particular, AMPK–DAPK3–ULK1 signaling was studied in differentiating adipocytes, and the kinase findings cannot automatically be generalized across whole tissues [115]. Increased UCP1 and circulating norepinephrine in another mouse study were compatible with sympathetic involvement, but β-adrenergic necessity was not demonstrated by receptor blockade, as the authors subsequently acknowledged [116]. A meta-analysis of nine randomized trials involving 525 participants found no significant effect on body weight (mean difference −0.35 kg; 95% CI, −2.03 to 1.33), BMI, waist circumference, or waist-to-hip ratio (WHR) [117]. A later double-blind crossover trial randomized 41 adults with nonalcoholic fatty liver disease (NAFLD) to quercetin capsules at 500 mg/day or placebo for 12-week treatment periods; 36 completed the study. Magnetic resonance imaging (MRI)-measured hepatic fat decreased during quercetin treatment, and mean weight changes were −1.5 kg with quercetin and −0.2 kg with placebo. Hepatic-fat change correlated with weight change, so a weight-independent hepatic benefit was not established [112]; this is a preliminary finding from one trial, not a pooled estimate of hepatic-fat efficacy. Human pharmacokinetic evidence identifies chemical form, formulation, and food matrix as determinants of exposure [118]; its observations support attention to circulating species, but do not show that low bioavailability caused the neutral weight findings. The current evidence separates a plausible experimental mechanism and a population-specific liver-fat signal from unconfirmed sustained adiposity benefit [112,117,118].

### 5.2. Chlorogenic Acids

CGAs comprise related hydroxycinnamic-acid esters rather than one interchangeable intervention. Coffee is a prominent studied source, but artichoke, cardoon, and blueberries also contain chlorogenic or related caffeoylquinic acids [119,120,121]. Artichoke by-products can yield extracts enriched in mono- and dicaffeoylquinic acids, with composition dependent on the material and extraction process [121]. Chemical occurrence and extractability establish a source of compounds, not clinical equivalence to coffee preparation or an obesity treatment. CGA reduced weight gain and altered hepatic lipid-metabolism measures in high-fat-fed mice [122], while brown-adipocyte experiments found changes in glucose handling, mitochondrial function, and proton leak [91]. A later mouse study combined transcriptomics, metabolomics, and fecal transplantation and reported transferable metabolic changes [123]; its findings remain model-specific. Human coffee studies identify multiple parent and conjugated or microbial metabolites, which limits extrapolation from experiments using only the unconjugated parent compound [26].

In a 12-week trial of 150 adults with overweight, coffee containing 369 mg/day total CGAs reduced computed tomography (CT)-measured visceral and total abdominal fat compared with coffee containing 35 mg/day. Caffeine was closely matched at approximately 37–38 mg/day, but other coffee constituents also differed [113]. A 2023 meta-analysis of three small, heterogeneous green-coffee/CGA trials reported a weight difference of −1.30 kg (95% CI, −2.07 to −0.52), with low certainty in the source assessment [124]. This estimate cannot be assigned to all CGA isomers or preparations; a component-separated six-month trial in adults with T2D and fatty liver found no significant benefit from isolated CGA at 200 mg/day on hepatic fat assessed by controlled attenuation parameter (CAP) or on insulin resistance [114]. A coffee crossover study with 38 completers found a small body-fat-percentage difference without significant weight loss, and differing caffeine exposure limited attribution [125].

Non-coffee trials broaden the context without resolving compound attribution; a 12-week trial of a supplement containing artichoke and bergamot extracts, astaxanthin, chromium, and folate enrolled 90 adults with suboptimal cholesterol and excluded obesity. It improved selected lipids and a fatty-liver index, not directly imaged liver fat [126]. A more recent pilot in 40 bariatric-surgery candidates reported lower CAP and liver size with artichoke leaf extract over six weeks, with an increase in transaminases; the final three weeks also included a liver-reduction diet [127]. Neither study establishes that isolated CGA caused the effects, and agro-industrial extracts require their own characterization and clinical evaluation.

### 5.3. Epigallocatechin-3-Gallate

EGCG is a major gallated green-tea catechin. Experimental reports include pancreatic lipase inhibition, reduced weight gain in high-fat-fed mice, and altered adipogenic and AMPK/ACC-associated responses [46,128]; autophagic responses differed between differentiating cells and adipose tissue [128]. The proposed catechol-O-methyltransferase (COMT) explanation for human thermogenesis also remains uncertain: an acute study in 24 volunteers found no inhibition of erythrocyte COMT after EGCG administration [129]. Most clinical efficacy evidence concerns green-tea preparations; a 2025 meta-analysis estimated differences of −0.74 kg in weight (95% CI, −0.97 to −0.51), −0.29 kg/m^2^ in BMI (−0.38 to −0.19), and −1.04 cm in waist circumference (−1.55 to −0.53) [130]. Also, in a 12-week trial of 60 adults, an extract providing more than 560 mg/day EGCG and 280–450 mg/day caffeine did not significantly alter fecal fat excretion, resting energy expenditure, or body composition [54]. A 2026 trial of catechins plus inulin likewise found no significant between-group difference in abdominal-bioimpedance-estimated visceral fat, despite a secondary HOMA-IR improvement [131]; combined products and distinct outcomes therefore cannot be treated as evidence of EGCG-specific fat loss.

Exposure also changes the safety interpretation; the European Food Safety Authority (EFSA) identified increased transaminases in trials using supplemental EGCG doses of at least 800 mg/day and could not establish a universally safe supplemental dose [27]. This observation does not guarantee safety below that level. Traditional infusions, concentrated extracts, caffeine co-exposure, and administration with or without food produce different exposure contexts [27,132]. Current evidence supports only preparation-specific, small average-weight effects for some green-tea products; the sustained efficacy of isolated EGCG remains unresolved [54,130].

### 5.4. Berberine

Berberine is an isoquinoline alkaloid evaluated here as a supplemental intervention. In muscle cells and isolated mitochondria it inhibited respiratory complex I, providing a basis for energy-sensing responses [133]. Adipocyte and mouse studies implicated SIRT1 in glucose uptake and inflammatory regulation, while another mouse study linked AMPK/SIRT1 responses to PPARγ deacetylation and thermogenic remodeling [90,134]. Clinical results depend on the endpoint. PREMOTE randomized 409 adults with T2D and identified glycemic, microbiome, and bile-acid changes; it did not establish microbiome-mediated adiposity reduction [29]. A 2026 meta-analysis of 23 trial reports estimated weight and BMI differences of −0.88 kg (95% CI, −1.36 to −0.39) and −0.48 kg/m^2^ (−0.89 to −0.07), respectively [135]. In contrast, BRAVO randomized 337 adults with obesity and MASLD without diabetes to berberine hydrochloride at 1 g/day or placebo for six months, alongside lifestyle intervention. It found no significant benefit in visceral or hepatic fat or body weight [28]; lipid and inflammatory secondary findings were exploratory because the study did not control for multiplicity. A separate 2026 trial reported improved bioimpedance-derived fat mass with a combination of berberine hydrochloride, white-mulberry leaf extract, and chromium picolinate [136]. Without single-component arms, the results cannot be attributed to berberine alone or used to establish synergy. Together, these studies support outcome- and preparation-specific interpretation, with modest pooled weight effects and discordant evidence for directly assessed fat depots [28,135,136].

## 6. Context from Other Phytochemical Interventions

Human evidence beyond the four focal compounds also depends on preparation and outcome. A network meta-analysis of 111 randomized trials (6171 participants; 18 nutraceutical interventions) reported modest weight reductions with variable certainty [137]. It included non-phytochemical products, so its rankings do not directly compare the selected phytochemicals. Clinical interpretation depends on the between-group estimate, uncertainty, follow-up, reproducibility, and chemical identity [138,139]. Table 2 summarizes the four examples and four additional groups, including representative doses, formulations, and adiposity outcomes.

Capsaicin-related interventions illustrate the difference between short-term physiological activity and weight-loss efficacy. A trial of a standardized capsaicinoid formulation reported changes in energy-expenditure measures [140], but a meta-analysis found only a small pooled body-weight difference (−0.51 kg; 95% CI, −0.86 to −0.15), with substantial heterogeneity [141]; in that analysis, neither the capsaicin-only nor the multicomponent subgroup showed a statistically significant pooled weight reduction. A separate capsinoid-focused meta-analysis found no significant overall effects on body weight, fat mass, or visceral fat area [142].

Saffron findings also depend on the measured outcome. An early trial reported reduced self-recorded snacking with a proprietary extract [143]. A 2022 meta-analysis found no significant overall change in body weight [144], whereas an updated 2026 analysis of 28 trials estimated a small reduction (−1.09 kg; 95% CI, −2.16 to −0.02) [146]. The latter confidence interval approaches no effect, and the trials differ in preparation and population. A separate 2025 synthesis found improvements in selected glycemic and blood-pressure outcomes without significant changes in BMI or waist circumference [146]. Appetite-related, metabolic, and anthropometric effects should therefore be considered separately.

Anthocyanin interventions show a similar distinction. A 2025 meta-analysis of 29 trials (2006 participants) found no significant pooled body-weight effect (−0.12 kg; 95% CI, −0.45 to 0.21), despite improvements in selected lipid and glycemic markers [147]. A 2026 synthesis reported vascular-function benefits in largely healthy populations without overall anthropometric benefits [148], while a 2025 review found no significant pooled changes in IL-1β, TNF-α, or IL-6 [149]. By contrast, phytosterols and phytostanols consistently lower LDL cholesterol but show no significant pooled effect on body weight or fat mass [151]. These distinctions favor outcome-specific claims rather than general labels of anti-obesity efficacy.

Sulforaphane illustrates why mechanistic plausibility and metabolic outcomes remain separate from weight-loss efficacy. In a 2025 trial, 89 adults with prediabetes were randomized to broccoli-sprout extract providing 150 μmol/day sulforaphane or placebo for 12 weeks. Among participants with full follow-up, the fasting-glucose difference was −0.2 mmol/L, smaller than the prespecified 0.3 mmol/L target, and BMI did not differ between groups [151]. This clinical example complements the NRF2-dependent mouse findings without establishing a human anti-adiposity effect [45,151].

These examples cover interventions with human studies; they cannot support a class-wide conclusion about all phytochemicals, particularly compounds for which human exposure or adiposity trials are absent. For less-studied candidates, absence of human evidence is an evidence gap rather than proof of no biological activity or no possible benefit. Across the evaluated products, appetite, glycemic, lipid, vascular, and inflammatory outcomes remain distinct from changes in total, visceral, or hepatic fat [138,139].

## 7. Exposure and Model Dependence of Experimental Findings

### 7.1. Exposure Plausibility in Cellular Studies

Cellular effects are interpretable only in relation to the tested chemical species, concentration, duration, and assay conditions. Nominal medium concentration may differ from available exposure because of binding, instability, or adsorption; viability and assay interference also affect interpretation [152,153]. Expression or phosphorylation changes, docking, and omics enrichment identify hypotheses unless supported by functional measurements and appropriate perturbation [153,154]; its principles explain specific uncertainties in Section 5 rather than providing a universal reporting checklist.

For quercetin, the 10 μM parent-compound exposure used in a differentiating-adipocyte experiment is distinct from the oral 500 mg/day clinical regimen and from circulating quercetin conjugates [112,116,118]. For CGA, brown-adipocyte experiments do not show that coffee-derived metabolites reproduce the same mitochondrial effects in vivo [26,91,155]. For EGCG, the chemical species matter: after a beverage containing 135 mg EGCG, a human pharmacokinetic study measured both unconjugated EGCG and EGCG-4″-sulfate at submicromolar peak concentrations [156]. These observations are incompatible with assuming that a swallowed daily dose directly specifies the concentration reaching adipocytes. For berberine, low and variable systemic exposure after an oral 1000 mg dose in healthy adults does not establish human adipose complex-I or SIRT1 engagement [133,134,157]. Luminal effects remain possible despite low plasma exposure, but require their own physiological evidence.

### 7.2. Animal Models, Dose Translation, and Thermogenic Context

Administration and model context further qualify the comparison: quercetin at 0.05% of mouse diet for nine weeks [116], CGA at 0.02% of a high-fat diet are not direct equivalents of the oral human regimens in Figure 2 [122]. Berberine thermogenesis experiments used gavage doses of 25 or 100 mg/kg/day for six weeks, including animals housed at 20 ± 2 °C [90]. Body-surface-area conversion alone cannot demonstrate matching parent-compound or metabolite exposure, especially when diet incorporation, gavage, formulation, and dosing frequency differ [152,158]. Prevention of weight gain also differs from treatment of established disease; changes in intake, fat and lean mass, feeding schedule, and housing temperature can influence apparent efficacy [69,158,159,160,161]. Pair-feeding can clarify intake-related effects but introduces dependence between paired animals and does not guarantee identical actual consumption [69]. Below-thermoneutral housing increases heat-production demands; an experimental transition from 24 °C to 30 °C reduced energy expenditure and brown-adipose UCP1 [161]. Accordingly, mouse UCP1 induction alone cannot establish clinically useful thermogenesis with quercetin, CGA, EGCG, or berberine.

## 8. Translation to Human Interventions

The evidence in Section 5 contains several incomplete links: administered product-to-tissue exposure, exposure to functional target engagement, and target-related change to a clinically relevant outcome.

### 8.1. Bioaccessibility, Pharmacokinetics, and Active Metabolites

The amount ingested is not a proxy for the chemical species reaching a tissue. Release from food or dosage form, intestinal metabolism, conjugation, microbial transformation, and clearance affect exposure [24]. Quercetin formulations differ in absorption [118,162]; CGA-rich coffee produces multiple metabolites, with evidence for colonic contributions [26,155]; EGCG occurs alongside sulfate metabolites [156]; and berberine exposure varies with sex and aspects of CYP2D6 metabolism [157]. These compound-specific observations do not account for all discrepancies between trials, nor do they show that increased bioavailability improves weight loss.

### 8.2. Chemical Standardization and Formulation

Chemical characterization is necessary to determine whether two studies evaluated comparable interventions. The Phytochemical Characterisation of Medicinal Plant (ConPhyMP) guidance describes botanical authentication, plant part, extraction, quantified markers, chemical fingerprints, and batch information for plant extracts [138]. This is directly relevant to quercetin aglycone versus enriched extracts, individual CGA isomers versus coffee mixtures, EGCG versus total catechins with caffeine, and berberine salts versus multicomponent products. A micellar quercetin pilot showed formulation-dependent circulating exposure, but did not test superior weight-loss efficacy or chronic safety [162]. Likewise, a positive result with an artichoke mixture or berberine combination cannot identify the active component without a design that can separate its effects [126,136].

### 8.3. Safety and Phytochemical–Drug Interactions

Safety is product-, exposure-, and duration-specific. For EGCG, concentrated-extract liver-enzyme findings and an exploratory genotype analysis of a high-dose trial do not establish a safe lower dose or a validated genotype-based prescribing rule [27,163]. For berberine, a controlled probe-drug study using 300 mg three times daily for two weeks found reduced CYP2D6, CYP2C9, and CYP3A4 activity [164]; this supports potential pharmacokinetic interactions under the tested conditions, not the same interaction for every phytochemical.

BRAVO’s comparable adverse-event incidence is also conditional: a 30-day berberine run-in excluded participants with intolerance or adherence below 80% before randomization [28]. Thus, the randomized safety comparison applies to a selected group of tolerators over six months. The short quercetin liver-fat trial and CGA trials likewise do not establish safety for indefinite use [112,113,114]. The transaminase increase in the artichoke pilot reinforces the need to interpret food-derived and concentrated-extract exposures separately [127]. Concomitant medications and actual adverse-event ascertainment remain essential to product-specific interpretation [35,139].

### 8.4. Clinical-Trial Design and Outcome Selection

Outcome choice changes the conclusion. Quercetin’s MRI liver-fat signal arose in a small NAFLD crossover trial, whereas its pooled weight estimate was neutral [112,117]. CGA-enriched coffee and isolated-CGA capsules differed in matrix, population, fat depot, and assessment method [113,114]. Green-tea pooled weight effects did not establish EGCG-specific efficacy [130], and a catechin–inulin combination showed a secondary insulin-resistance signal without a significant visceral-fat effect [131]. Berberine’s small pooled weight estimate coexisted with neutral BRAVO adiposity outcomes [28,135]; these discrepancies favor interpretation of between-group effects and uncertainty within each study, with weight, fat depots, lean mass, and metabolic surrogates kept distinct. Duration, co-interventions, attrition, multiplicity, and overlap among trials across meta-analyses further limit comparisons [139].

### 8.5. Variation in Response and Microbiome Findings

Variation in metabolism, diet, medications, and microbial function is biologically plausible, but no validated responder rule currently follows from the evidence reviewed here. Quercetin formulation studies and berberine pharmacokinetics describe exposure variability, not patient-selection algorithms [118,157,162]. CGA microbial metabolism and EGCG sulfation further alter the chemical species measured [26,155,156]. Fecal-transplantation/fiber data and standardized-meal studies provide contextual examples of response prediction, but they do not validate microbial signatures for phytochemical-induced fat loss [111,165]. In PREMOTE and the sulforaphane trial, microbiome associations concern specific preparations and glycemic outcomes [29,151]. A personalized-diet trial also illustrates that predictive information does not necessarily improve weight loss [166]. External validation and demonstration of clinical utility remain separate questions from identifying a statistical association [167,168]. Table 3 summarizes these sources of response heterogeneity, along with the compound-specific translational limitations discussed above.

## 9. Strengths and Limitations

This review integrates experimental mechanisms, human exposure, formulation, efficacy, and safety around four chemically distinct examples. It explicitly separates model-level causal evidence from associative markers and distinguishes anthropometric effects from glycemic, lipid, or inflammatory outcomes. Including favorable and neutral findings highlights where interpretation shifts by preparation, population, or fat depot measured [28,54,112,113,114,117,124,125,130,135]. Its contribution is a critical account of these translational links, rather than a treatment ranking.

Several limitations constrain the synthesis. Literature selection was purposive and favored compounds with human studies, limiting the representativeness of the review for less-studied phytochemicals. Search coverage was not exhaustive: publication-type indexing may omit relevant reports, and retrieval from the broad food-source query was limited. Detailed records of the original Google Scholar searches were unavailable, and an independent duplicate-screening process was not documented. The synthesis does not include a new study-level risk-of-bias assessment or certainty grading. These limitations preclude an exhaustive inventory or a formal comparison of intervention efficacy.

The underlying studies differ in chemical composition, formulation, dose, experimental model, participant characteristics, co-interventions, duration, and outcome. Some reports could be assessed only from their abstracts, limiting appraisal of their methods and results. Meta-analyses may include overlapping trials, and short follow-up and incomplete safety reporting restrict conclusions about chronic use. Publication bias and selective reporting remain possible. The studies also do not consistently characterize participants according to ABCD severity or assess functioning and quality of life. The available evidence therefore leaves the durability and wider clinical relevance of the observed effects uncertain.

## 10. Conclusions

For the interventions evaluated, human adiposity findings were small, neutral, or dependent on the preparation and population studied. Quercetin showed neutral pooled anthropometric results alongside a hepatic-fat signal from one small crossover trial that requires replication. Findings from CGA-rich coffee and green-tea preparations do not establish the efficacy of isolated CGA or EGCG. Berberine showed a small pooled weight difference, while a six-month trial found no significant benefit for visceral fat, hepatic fat, or body weight in adults with obesity and MASLD without diabetes. The central translational gap is whether a well-characterized preparation can achieve relevant human exposure and produce sustained improvements in adiposity or ABCD-related health outcomes at a tolerable dose. Pathway activity and short-term changes in metabolic markers do not resolve that question. Within comprehensive ABCD care, clinical value depends on demonstrated benefit in complications, functioning, quality of life, or sustained adiposity reduction, considered alongside product-specific adverse effects and interactions. The evidence reviewed here does not yet establish routine use of these four interventions as treatments for sustained adiposity reduction.

## Figures and Tables

**Table 1:** Tissue-specific pathways regulating energy balance and metabolic homeostasis.

Biological Process	Key Molecular Components	Main Physiological Role	References
Intestinal digestion	Pancreatic lipase; pancreatic α-amylase. Brush-border α-glucosidases: maltase-glucoamylase and sucrase-isomaltase.	Lipid and carbohydrate hydrolysis; nutrient availability.	[46,47,48,51,52,53]
Gut–brain signaling and appetite	Leptin–STAT3; insulin; ghrelin; GLP-1/PYY/CCK. Hypothalamic POMC and NPY/AgRP.	Integration of energy-status and meal signals through vagal–brainstem and hypothalamic circuits.	[55,56,57,58,59,60,61,62,67]
Adipogenesis and lipogenesis	Adipogenesis: C/EBPβ–PPARγ/C/EBPα. Lipogenesis: SREBP-1c–ACC/FASN. Lipid storage: PPARγ–LPCAT3.	Adipocyte differentiation, fatty-acid synthesis, and storage; LPL supplies fatty acids from circulating lipoprotein triglycerides.	[43,72,73,76,77,78]
Lipolysis and fatty-acid oxidation	Lipolysis: ATGL/HSL/MGL. Oxidation: AMPK–ACC–malonyl-CoA–CPT1; SIRT1–PGC-1α–PPARα.	Triglyceride mobilization and regulation of mitochondrial fatty-acid oxidation.	[42,79,80,81,82,83,84,85]
Thermogenesis and browning	β-Adrenergic receptors–cAMP–PKA; p38 MAPK. PGC-1α/PRDM16; UCP1. AMPK–SIRT1-associated PPARγ deacetylation.	Thermogenic adipocyte programming and mitochondrial energy dissipation as heat.	[86,87,88,89,90]
Insulin signaling and glucose homeostasis	Insulin receptor–IRS–PI3K–Akt–TBC1D4/AS160; GLUT4. Adiponectin–AMPK.	Glucose uptake in muscle and adipose tissue; fatty-acid oxidation.	[43,94,95,96,97]
Adipose inflammation and redox regulation	Inflammation: TLR4–MyD88–IKKβ/NF-κB; JNK; NLRP3. Redox adaptation: KEAP1–NRF2–ARE. AMPKα1/SIRT1. Sulforaphane/glucoraphanin as a specific NRF2 example.	Cytokine production, immune-cell responses, and adaptation to oxidative stress.	[44,45,98,99,100,101,102,103]
Microbiota, intestinal barrier, and bile acids	SCFAs–FFAR3; bile acids–TGR5. Indolepropionic acid–tuft-cell IL-25.	Microbial signaling affecting feeding, thermogenesis, and intestinal-barrier integrity.	[104,105,106,107,108]

Abbreviations: ACC, acetyl-CoA carboxylase; AgRP, agouti-related peptide; Akt, protein kinase B; AMPK, AMP-activated protein kinase; ARE, antioxidant response element; ATGL, adipose triglyceride lipase; cAMP, cyclic adenosine monophosphate; C/EBP, CCAAT/enhancer-binding protein; CCK, cholecystokinin; CoA, coenzyme A; CPT1, carnitine palmitoyltransferase 1; FASN, fatty-acid synthase; FFAR3, free fatty-acid receptor 3; GLP-1, glucagon-like peptide-1; GLUT4, glucose transporter type 4; HOMA2-IR, updated homeostatic model assessment of insulin resistance; HSL, hormone-sensitive lipase; IKKβ, inhibitor of nuclear factor κB kinase β; IL-25, interleukin-25; IRS, insulin receptor substrate; JNK, c-Jun *N*-terminal kinase; KEAP1, Kelch-like ECH-associated protein 1; LPCAT3, lysophosphatidylcholine acyltransferase 3; MAPK, mitogen-activated protein kinase; MGL, monoacylglycerol lipase; MyD88, myeloid differentiation primary response 88; NF-κB, nuclear factor κB; NLRP3, NLR family pyrin domain-containing 3; NPY, neuropeptide Y; NRF2, nuclear factor erythroid 2-related factor 2; PGC-1α, PPARγ coactivator 1α; PI3K, phosphoinositide 3-kinase; PKA, protein kinase A; POMC, pro-opiomelanocortin; PPARα/γ, peroxisome proliferator-activated receptor α/γ; PRDM16, PR domain-containing protein 16; PYY, peptide YY; SCFAs, short-chain fatty acids; SIRT1, sirtuin 1; SREBP-1c, sterol regulatory element-binding protein 1c; STAT3, signal transducer and activator of transcription 3; TBC1D4/AS160, TBC1 domain family member 4/Akt substrate of 160 kDa; TGR5, Takeda G protein-coupled receptor 5; TLR4, toll-like receptor 4; UCP1, uncoupling protein 1.

**Table 2:** Human evidence for selected phytochemicals and phytochemical-rich interventions.

Intervention	Representative Oral Dose and Duration	Adiposity Findings	Interpretation	References
Quercetin capsules or enriched preparations	Trials in the meta-analysis: 100–1000 mg/day, 2–12 weeks. Liver-fat trial: quercetin 500 mg/day, 12 weeks/period	Pooled weight −0.35 kg (95% CI −2.03 to 1.33). One small NAFLD trial reported MRI liver-fat and weight reductions	Pooled estimate neutral; single liver-fat trial is separate evidence	[112,117]
CGA-enriched coffee and isolated CGA	Coffee: 369 vs. 35 mg/day total CGAs, 12 weeks; caffeine approximately 37–38 mg/day. Isolated CGA: 200 mg/day, 6 months	Selected green-coffee/CGA trials: pooled weight −1.30 kg (−2.07 to −0.52), low certainty. Coffee trial reduced abdominal fat; isolated-CGA hepatic result NS	Matrix and isomer differences prevent molecule-specific attribution	[113,114,124,125]
Green-tea preparations and EGCG-containing extracts	Example: extract providing >560 mg/day EGCG plus 280–450 mg/day caffeine, 12 weeks	Green-tea pooled weight −0.74 kg (−0.97 to −0.51). Example trial: body composition and fecal fat NS	Estimate is not purified-EGCG efficacy; extract-related liver risk matters	[27,54,130]
Berberine preparations; BRAVO used hydrochloride	BRAVO: 500 mg twice daily, 6 months, with lifestyle intervention in both arms	Pooled weight −0.88 kg (−1.36 to −0.39). BRAVO: visceral fat, liver fat, and weight NS	Effects depend on endpoint and population; tolerability run-in limits safety generalization	[28,135]
Capsaicinoids and capsinoids	Capsifen example: 100 or 200 mg/day extract containing 2.1% capsaicinoids (2.1 or 4.2 mg/day), 28 days	Mixed capsaicin interventions: pooled weight −0.51 kg (−0.86 to −0.15). Capsinoid overall weight/fat effects NS	Example regimen is not a uniform pooled dose; compounds and mixtures differ	[140,141,142]
Saffron preparations and isolated crocin	Saffron-extract example: Satiereal 176.5 mg/day in two capsules, 8 weeks	2026 pooled weight −1.09 kg (−2.16 to −0.02); earlier synthesis neutral	Extract mass is not isolated-crocin dose; small borderline pooled effect	[143,144,145,146]
Anthocyanins in foods, extracts, or purified preparations	Source-reported range: 0.26–900 mg/day anthocyanins; 4 days–24 weeks	Pooled weight −0.12 kg (−0.45 to 0.21). Selected metabolic outcomes differed	Heterogeneous sources and exposure; biomarker benefit is not fat loss	[147,148,149]
Phytosterols and phytostanols in enriched foods or supplements	Updated lipid dose-response identified LDL-C effects at 0.5–1 g/day; this is not an adiposity-effective dose	No significant pooled weight or fat-mass effect; LDL-C reductions were consistent	Established cholesterol effect does not establish sustained adiposity reduction	[150]

Confidence intervals are 95% intervals. Abbreviations: CI, confidence interval; CGAs, chlorogenic acids; CT, computed tomography; EGCG, epigallocatechin-3-gallate; LDL-C, low-density lipoprotein cholesterol; MRI, magnetic resonance imaging; NAFLD, nonalcoholic fatty liver disease; NS, no statistically significant between-group effect.

**Table 3:** Compound-specific translational limitations and their consequences for interpreting efficacy.

Translational Issue	Example from the Selected Compounds	Consequence for Interpretation	References
Chemical identity and co-constituents	CGA-rich coffee, catechins plus inulin, and berberine combinations contain components beyond the named molecule.	A product effect cannot be assigned to an isolated constituent without a suitable comparison.	[113,131,136,138]
Measured chemical exposure	Quercetin conjugates, CGA microbial metabolites, EGCG sulfate, and variable berberine exposure differ from nominal cell-culture exposure.	A plausible systemic mechanism depends on the actual chemical species and concentration at the site of action.	[26,118,155,156,157,162]
Mechanism and physiological function	Quercetin or berberine thermogenic markers in mice; EGCG-associated AMPK/ACC and autophagy changes.	Expression and phosphorylation are not equivalent to human target engagement or whole-body fat loss.	[90,116,128,153,154]
Administration and animal environment	Dietary quercetin/CGA versus gavaged berberine; rodent housing below thermoneutrality.	Dose conversion alone cannot bridge route, metabolism, intake, and thermal demand.	[69,90,116,122,128,158,159,160,161]
Clinical endpoint and preparation	Quercetin liver-fat signal; CGA coffee/capsule differences; green-tea pooled effects; neutral BRAVO fat depots.	Each finding remains tied to its population, comparator, duration, and primary or secondary endpoint.	[28,112,113,114,117,130,135,139]
Safety and interactions	EGCG extract transaminases; berberine CYP interactions and tolerance-enriched BRAVO randomization.	Short-term tolerability or selected-tolerator data do not establish unrestricted long-term safety.	[27,28,163,164]
Response heterogeneity	Quercetin formulation, berberine disposition, and exploratory microbiome associations.	Candidate predictors remain unvalidated for choosing a phytochemical to reduce adiposity.	[29,118,151,157,162]

Abbreviations: ACC, acetyl-CoA carboxylase; AMPK, AMP-activated protein kinase; CGA, chlorogenic acid; CYP, cytochrome P450; EGCG, epigallocatechin-3-gallate.

## Data Availability

No new datasets were generated in this study. All information presented in this narrative review was obtained from previously published studies cited in the manuscript.
